# Mastitomics, the integrated omics of bovine milk in an experimental model of *Streptococcus uberis* mastitis: 1. High abundance proteins, acute phase proteins and peptidomics†
†Electronic supplementary information (ESI) available. See DOI: 10.1039/c6mb00239k



**DOI:** 10.1039/c6mb00239k

**Published:** 2016-07-14

**Authors:** Funmilola Clara Thomas, William Mullen, Riccardo Tassi, Adela Ramírez-Torres, Manikhandan Mudaliar, Tom N. McNeilly, Ruth N. Zadoks, Richard Burchmore, P. David Eckersall

**Affiliations:** a Institute of Biodiversity Animal Health and Comparative Medicine , University of Glasgow , Bearsden Road , Glasgow , G61 1QH , UK . Email: david.eckersall@glasgow.ac.uk; b Institute of Cardiovascular and Medical Sciences , University of Glasgow , University Avenue , Glasgow , UK; c Moredun Research Institute , Pentlands Science Park/Bush Loan , Penicuik , UK; d Mosaique Diagnostics GmbH , Rotenburger Str. 20 , D-30659 Hannover , Germany; e Glasgow Polyomics , College of Medical , Veterinary and Life Science , University of Glasgow , Glasgow , UK

## Abstract

A peptidomic investigation of milk from an experimental model of *Streptococcus uberis* mastitis in dairy cows has incorporated a study of milk high abundance and acute phase (APP) proteins as well as analysis of low molecular weight peptide biomarkers. Intramammary infection (IMI) with *S. uberis* caused a shift in abundance from caseins, β-lactoglobulin and α-lactalbumin to albumin, lactoferrin and IgG with the increase in lactoferrin occurring last. The APP response of haptoglobin, mammary associated serum amyloid A3 and C-reactive protein occurred between 30–48 hours post challenge with peak concentrations of APPs at 72–96 hours post challenge and declined thereafter at a rate resembling the fall in bacterial count rather than the somatic cell count. A peptide biomarker panel for IMI based on capillary electrophoresis and mass spectrometry was developed. It comprised 77 identified peptides (IMI77) composed mainly of casein derived peptides but also including peptides of glycosylation dependent cell adhesion molecule and serum amyloid A. The panel had a biomarker classification score that increased from 36 hour to 81 hour post challenge, significantly differentiating infected from non-infected milk, thus suggesting potential as a peptide biomarker panel of bovine mastitis and specifically that of *S. uberis* origin. The use of omic technology has shown a multifactorial cross system reaction in high and low abundance proteins and their peptide derivatives with changes of over a thousand fold in analyte levels in response to *S. uberis* infection.

## Abstract 2

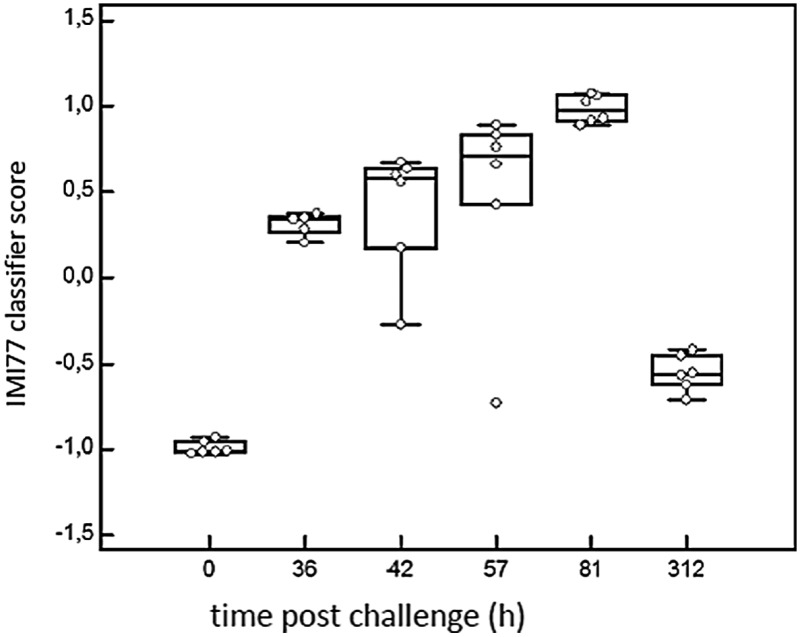
Acute phase proteins and a group of 77 peptides in a biomarker panel increase in milk during bovine mastitis caused by a *Streptococcus uberis* infection of mammary glands.

## 1. Introduction

Mastitis, mostly caused by bacterial infection of the mammary gland, is the major infectious disease problem in dairy cows, being estimated to cost the global dairy industry €16–26 billion per annum based on a global dairy cow population of 271 million dairy cows (www.dairy.ahdb.org.uk, accessed March 2016) and a cost to farmers of €61–97 per animal.^1^ The early detection of intra-mammary infections (IMI), the main cause of mastitis, would be greatly beneficial in allowing early treatment and prevention of onward transmission of disease. Furthermore early characterisation of the bacterial species causing mastitis would allow more targeted chemotherapy, which may help to reduce inappropriate use of antibiotics.^2^ The last decade has shown a major increase in the use of omics technologies in experimental biology and human disease investigations, but, with the exception of genomics, the application of advanced analytical technologies such as proteomics and metabolomics has been limited in studies of animal health and disease. This is undergoing change.^3^ This paper is the first of a series of three in which protein and metabolite alteration in the composition of milk during bovine mastitis was investigated with the aim of characterising the molecular biosystem of milk to increase our understanding of the pathology of the disease and to identify potential biomarkers for early detection of IMI.

In this series of studies, changes in milk during mastitis were investigated utilising an established experimental model of the disease^4^ induced by *Streptococcus uberis* (*S. uberis*) which is one of the most prevalent causes of bovine mastitis in the United Kingdom^5–7^ and other countries.^6–9^ In the first paper, we focus on high abundance proteins, acute phase proteins^10^ and quantitative peptidomics.^11^ In the subsequent paper, a label free quantitative proteomic method will be used to monitor changes in higher *M*_w_ proteins of milk,^12^ and in the final paper of the series, the alteration of low *M*_w_ metabolites will be described.^13^ All investigations used milk samples from an experimental model of *S. uberis* mastitis used for the investigation of host immune responses in milk.^4^ This has previously revealed changes in concentrations of cytokines such as TNFα and interleukins 1-β and 6, which are associated with induction of the acute phase response,^14–16^ as well as recruitment of lymphocytes (CD4, CD8 and γδ T cells) and polymorphonuclear cells into the milk.^4^

The high abundance proteins in healthy milk consist largely of the caseins, β-lactoglobin and α-lactoglobin^17^ and reduction in these major proteins in milk due to IMI have been documented,^18,19^ as well as increases in albumin, lactoferrin and immunoglobulins.^20,21^ However there has been little investigation of the time course of changes in these high abundance proteins particularly in relation to changes in the low abundance proteins such as acute phase proteins (APP) in milk.

Acute phase proteins are serum proteins which increase (or decrease) in concentration by over 25% following stimulation by pro-inflammatory cytokines such as TNFα and IL6, and APP are now recognised as also being elevated in milk during mastitis.^22^ Haptoglobin (Hp) and mammary associated serum amyloid A3 (MSAA3), the isoform of SAA synthesised and secreted by the mammary epithelial cells are recognised as milk APP. For example, Pedersen and others^23^ studied the early inflammatory responses of the host to an experimental *S. uberis* infection and showed that infection causes a rise in milk Hp and MSAA3. Previous studies in an experimental model of *Staphylococcus aureus* (*S*. *aureus*) induced mastitis have also demonstrated that measuring APP could be useful in identification of the inflammatory response to the mammary infections.^10^ Although several recent studies on APP in milk during mastitis have focussed on Hp and SAA, some investigations have identified a possible value of bovine milk C-reactive protein (CRP) as a biomarker of bovine mastitis.^24–27^ However, variation of CRP during the course of an experimental infection has not been previously reported. In addition, APP profiles have been described during the onset of infection, but seldom during resolution of IMI. Knowledge of the change in concentration during resolution of infection is crucial for assessment of the diagnostic specificity of APP as an indicator of IMI.

While there have been several proteomic investigations of milk during mastitis^20,28–30^ the lower *M*_w_ peptides of milk have had less investigation. Our earlier study of the peptidome of milk during clinical mastitis, caused by *S. aureus* and *Escherichia coli*, indicated that analyses using a peptide biomarker panel could have potential in diagnosis of the disease^11^ but the milk peptidome has not been monitored for changes over the course of an experimental infection. Biomarker discovery using a combination of capillary electrophoresis and mass spectroscopy (CE-MS) has enabled the identification of peptide panels which are used in diagnostic procedures for human diseases^31^ and have the ability to be applied to diseases of livestock.^32^

Therefore the aim of this study is to identify the effects of *S*. *uberis* mastitis on the molecular pathophysiology of (a) high abundance milk proteins, (b) the APP in the low abundance milk proteins and (c) low *M*_w_ peptides (<25 kD) in milk during IMI. The research described here is the first of three linked mastitomic studies^11,12^ which along with clinical and immunological data of the same sample sets^4^ aims to contribute to an integrated systems biology approach to increase our understanding of bovine mastitis.

## 2. Methods

### 2.1 Experimental challenge model of *S. uberis* mastitis

Milk samples were obtained from an intramammary challenge study of a single udder quarter from each of six healthy Holstein cows using a putative host adapted strain of *S. uberis*, strain FSL Z1-048. Full details of the procedure and the results of clinical evaluation of infected cows as well as laboratory investigation of these milk samples such as for microbiology, somatic cell count (SCC), cytokines and lymphocyte ratios have been previously reported.^4^ The milk samples were stored at –20 °C in the period between the analyses reported in Tassi *et al.* ^4^ and the investigation described here. Samples were obtained at 19 time points from each challenged quarter comprising 0, 6, 12, 18, 24, 30, 36, 42, 48, 57, 72, 81, 96, 120, 144, 168, 192, 240 and 312 hours (h) post challenge (PC) and at 7 time points including 0, 12, 36, 57, 96, 192 and 312 h PC, from the control quarters (*n* = 1 per cow) that were infused with 2 ml sterile phosphate buffered saline (PBS). The timings were designed for collection at every 6 hours for the first 2 days; from 2 to 11 d PC, milk samples were collected twice a day; and from 11 to 13 d PC once a day. Skimmed milk was prepared by centrifuging 50 ml of milk at 2800 × *g* at 4 °C for 20 minutes (min). The fat layer was discarded and the supernatant was transferred to a new 50 ml Falcon tube. Centrifugation was repeated and the supernatant was stored at –20 °C. All animal experiments were conducted at the Moredun Research Institute (Penicuik, UK) with approval of the Institute’s Experiments and Ethical Review Committee in accordance with the Animals (Scientific Procedures) Act 1986.^4^

### 2.2 High abundance milk proteins: one dimensional electrophoresis

Prior to gel electrophoresis, protein concentration was determined using the Bradford protein assay with bovine serum albumin as standard (BSA; Sigma-Aldrich, USA). Sodium dodecyl sulphate polyacrylamide gel electrophoresis (SDS-PAGE) was performed on 4–15% gradient polyacrylamide gels in a Criterion electrophoresis system (BioRad Ltd, Hemel Hempstead, UK) as previously described.^33^ Samples of milk taken at each time point were separated by SDS-PAGE. The identity of protein in the SDS-PAGE bands was determined in a reference gel by analysis of milk from a healthy cow and a cow with mastitis run under the identical conditions, followed by LC-MS/MS. Protein bands were excised and processed^33^ prior to analysis at Glasgow Polyomics on a nanoflow uHPLC system (Thermo RSLCnano) and electrospray ionisation (ESI) mass spectrometry (MS) on an Amazon ion trap MS/MS (Bruker Daltonics). MS data were processed using Data Analysis software (Bruker) and the automated Matrix Science Mascot Daemon server (v2.1.06). Protein identifications were assigned using the Mascot search engine to interrogate protein sequences in the NCBI databases restricting the search to *Bos taurus* proteins.

### 2.3 Acute phase protein assays

Milk samples from all 19 time points (for challenged quarters; 7 time points for control quarters) were assayed for bovine Hp, MSAA3 and CRP. An in-house ELISA for bovine Hp using purified polyclonal rabbit anti-bovine Hp IgG (Life Diagnostics Inc, West Chester, Pennsylvania, USA) was carried out as described previously.^27^ Commercial ELISAs for SAA (Tridelta Development Ltd, Dublin Ireland) and bovine CRP (Life Diagnostics Inc, West Chester, Pennsylvania, USA) were used to quantify these proteins in milk from the *S. uberis* experimental model of mastitis as described previously.^27^

### 2.4 Peptidome analysis: sample preparation, CE-MS setup and data processing

Samples were prepared and run on capillary electrophoresis-mass spectrometry (CE-MS) with modifications to the methods described previously.^11^ Briefly, 0.1% PMSF was added to each milk sample. Aliquots of 150 μl were diluted with the same volume of 2 M urea, 100 mM NaCl, 10 mM NH_4_OH and 0.02% SDS. High *M*_w_ molecules were filtered with a cut-off >20 kDa Centrisart ultrafiltration tube (Sartorius, Germany) for 1 h, 3400 rpm, 4 °C. To discard urea and electrolytes, a NAP-5 column (GE Healthcare, Sweden) was used, equilibrated as recommended by the manufacturer. To elute the peptides from the column, 700 μl of the NH_4_OH were used. Protein concentration was determined by a bicinchoninic acid (BCA) assay, using BSA as standard. Aliquots were restored to a final concentration of 2 μg μl^–1^ prior to CE-MS analysis.

For the CE-MS analysis a Beckman Coulter P/ACE MDQ CE system (Fullerton, USA) was used. Before analysis, samples were centrifuged at 14 000 × *g* for 10 min at 4 °C. The peptides eluting from the CE were ionised using an electro-spray ionisation (Agilent Technologies, Palo Alto, CA, USA) which was grounded to achieve electric potential of 0, and the electro-spray interface potential of the microTOF mass spectrometer (Bruker Daltonics, Bremen, Germany) was set between –4 and –4.5 kV. The mass calibration of the microTOF was performed on a weekly basis using the standard protein/peptide solution (0.5 pmol μl^–1^) for CE-MS analysis. The acquisition of data and MS were automatically controlled by the CE *via* contact close-relays and MS spectra accumulated every 3 s, over a *m*/*z* range 350–3000 for 55 min.

MosaiquesVisu software was used to interpret the mass spectral ion peaks representing identical molecules at different charge states and thus, those signals were deconvoluted into single masses.^34^ The software automatically examined all mass spectra from a CE-MS analysis for signals with a signal-to-noise ratio of at least 4 present in three consecutive spectra. Additionally, the isotopic distribution was assessed, and charge was assigned on the basis of the isotopic distribution, as well as conjugated masses, with a probabilistic clustering algorithm. This operation resulted in a list wherein all signals that could be interpreted are defined by mass/charge, charge, migration time, and signal intensity (ion counts). Time-of-flight MS data were calibrated with Fourier transform ion cyclotron resonance MS data as reference masses applying linear regression. CE migration time was calibrated by local regression with 488 reference signals or “housekeeping polypeptides”. The obtained peak lists characterize each polypeptide by its molecular mass [Da], normalized CE migration time [min] and normalized signal intensity. All detected peptides were deposited, matched, and annotated in a Microsoft SQL database allowing further statistical analysis. For clustering, peptides in different samples were considered identical if mass deviation was <50 ppm. CE migration time was controlled to be below 0.35 minutes after calibration.

### 2.5 Peptides selection and statistical analysis

For the identification of potential IMI biomarkers, the normalized levels of cow milk peptides were compared between time point 0 h (non-infected or control group, *n* = 6) and time point 81 h (infected, *n* = 6). Only peptides that were detected with a minimal frequency of 4 of 6 in at least one of the diagnostic groups were considered for statistical analysis. Unadjusted P values were calculated for the comparison between the non infected and infected cow groups with the Wilcoxon rank-sum test followed by adjustment for multiple testing with the method described by Benjamini and Hochberg.^35^ Only peptides with a corrected *P* < 0.05 were considered significant.

The number of peptides with differential abundance was reduced to a support vector machine (SVM) classifier with 77 peptides (IMI77) by a take-one-out procedure. Sensitivity and specificity of the biomarker classifier in the discovery set, and 95% confidence intervals (95% IC) were calculated using receiver operating characteristic (ROC) plots (MedCalc versión 14.8.1, MedCalc Software, Belgium).

### 2.6 Liquid chromatography and mass spectrometry for peptide biomarker identification

In order to determine the sequences of significant biomarker polypeptides, LC-MS/MS peptide sequencing was carried out as previously described.^11^ Briefly, the milk extracts were analysed on a Dionex Ultimate 3000 RSLS nano flow system (Dionex, Camberly, UK). The samples were eluted with a gradient of solvent A: 0.1% formic acid and acetonitrile (98 : 2) *versus* solvent B: 0.1% formic acid and acetonitrile (20 : 80) starting at 5% B rising to 50% B over 100 min. The column was washed using 90% B before being equilibrated prior to the next sample being loaded.

The eluate from the column was directed to a Proxeon nano spray ESI source (Thermo Fisher Hemel, UK) operating in positive ion mode then into an Orbitrap Velos FTMS (Thermo Fisher Hemel, UK). The ionisation voltage was 2.5 kV and the capillary temperature was 250 °C. The mass spectrometer was operated in MS/MS mode scanning from 380 to 2000 amu.

Raw spectral data from LC-MS/MS analysis of the samples were uploaded to Thermo Proteome Discoverer 1.3. Only peptides with signal to noise ratio higher than 1.5 and belonging to precursor peptides between 380–6000 Da were considered. Peptide and protein identification was performed with the SEQUEST algorithm. An in-house database containing proteins from the latest version UniProt SwissProt database was compiled to include only *Bos taurus* and *S. uberis* entries. No enzyme cleavage was selected and oxidation of methionine and proline were chosen as variable modifications. Precursor mass tolerance was set at 5 parts per million (ppm) and 0.1 Da for MS/MS fragment ions. Resulting peptides and protein hits were further screened by excluding peptides with an error tolerance higher than 10 ppm and by accepting only those hits listed as high confidence by Proteome Discoverer software. Target false discovery rate (FDR) was 0.01 (strict) or 0.05 (relaxed).

## 3. Results

### 3.1 High abundance proteins

**Fig. 1:**
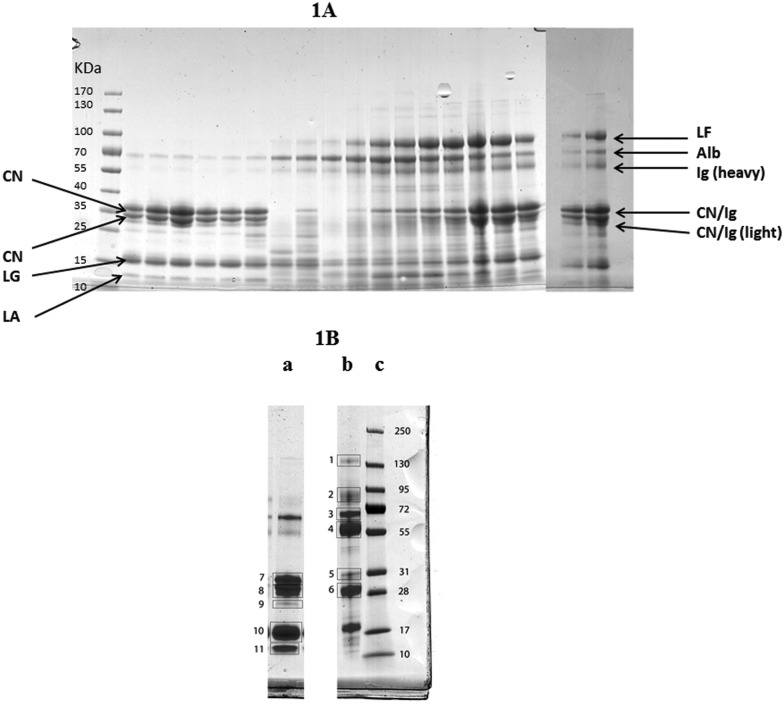
(A) One dimensional gel showing high abundance proteins from a mammary quarter challenged with *Streptococcus uberis* (panel A) (from left to right: size marker with band size in kDa, 0, 6, 12, 18, 24, 30, 36, 42, 48, 57, 72, 81, 96, 120, 144, 68, 192, 240 and 312 hours post challenge). Proteins were identified through comparison with results from reference samples shown in (B) with the main proteins shown here: LF = lactoferrin; Alb = albumin, Ig = Immunoglobulin; CN = casein; LG = lactoglobulin; LA = lactalbumin. (B) One dimensional gel showing (left to right) high abundance proteins from a healthy mammary quarter (a), high abundance proteins from a quarter with clinical mastitis of unknown etiology (b), and size marker with band sizes in kDa (c). Based on LC-MS/MS analysis (Table 1), bands were identified as (1) IgG heavy chain and ceruloplasmin; (2) lactoferrin, lactotransferrin precursor and serotransferrin precursor; (3) albumin and complement C3; (4) Ig heavy chain precursor and IgG heavy chain constant region; (5) Ig heavy chain precursor and light chain, alpha-S1-casein; (6) immunoglobulin lambda like polypeptide and light chain; (7) alpha-S1-casein and beta-lactoglobulin; (8) beta casein and component PP3; (9) beta-lactoglobulin and alpha-S1-casein; (10) beta-lactoglobulin; (11) alpha- and beta-lactoglobulin.

The alteration in the high abundance proteins of milk during the experimental infection with *S. uberis* is shown in Fig. 1A with milk protein from a single cow (cow 6) from 0 to 312 h PC separated by SDS-PAGE. Similar gels for samples from all cows are given in ESI† files (Fig. S1). The identity of the separated milk protein bands was determined by MS analysis of bands cut from a reference gel of healthy and mastitic milk (Fig. 1B) with the proteins identified listed in Table 1. Similar patterns of change after infection of the high abundance proteins of milk were obtained in samples of milk from all the infected quarters, though with some variation in the timing evident in Fig. S1 (ESI†). For instance the fall in the casein proteins at *M*_w_ 28–31 kDa was apparent in all cows but was first noticeable at 30 h (cow 2 & 3), 36 h (cow 1, 4, 6) or 42 h (cow 5) in different cows. Although the identity of most proteins in Fig. 1A and Fig. S1 (ESI†) was determined by comparison to the reference gel (Table 1) the identity of the proteins at *M*_w_ 28–31 kDa was less certain. The protein band at 31 kDa in healthy milk is α_s1_-casein and the protein at 28 kDa was β-casein, whereas in the mastitic milk both of these bands were IgG light chain. The protein bands at 28–31 kDa appearing from 72 h PC could be either caseins or IgG light chain. Overall the normal pattern of milk protein was found in the initial samples with αs_1_- and β caseins, β-lactoglobulin and α-lactalbumin predominating. Thereafter, taking sample 6 as an exemplar (Fig. 1A) these proteins are reduced between 30 and 81 h PC while there is an increase in albumin, lactoferrin (LF) and IgG heavy chain. Of these, an observable increase in albumin and IgG took place at 36 h PC with LF having a more delayed response. In comparing the albumin and LF protein bands, from 36–57 h PC the albumin band was more intense while from 96–192 h PC the LF band was more intense than the albumin (Fig. 1A). In the last sample taken (312 h PC) all of the high abundance proteins were still present, although infection had been resolved in the majority of quarters.^4^

**Table 1:** Milk proteins identified by LC-MS/MS after one dimensional SDS-PAGE separation of milk proteins

Band no.	Proteins	Protein ID	Mass	pI	Mowse score	Peptides	Sequence cover (%)
1	IgG heavy chain	gi|7547266	36 510	6.09	283	7	42
1	IgG heavy chain	gi|91982959	36 562	6.49	210	6	36
1	Ceruloplasmin	gi|296491101	121 901	5.68	90	9	9
2	Lactoferrin	gi|408928	80 113	8.73	1896	40	61
2	Lactotransferrin precursor	gi|30794292	80 002	8.69	1892	40	61
2	Serotransferrin precursor	gi|114326282	79 856	6.75	403	21	33
3	Albumin	gi|1351907	71 244	5.82	2449	47	68
3	Complement C3 isoform X1	gi|741932316	188 675	6.41	235	14	9
4	Ig heavy chain precursor	gi|108750	51 391	6.1	215	6	19
4	IgG2a heavy chain constant region, partial	gi|1699167	36 402	7.7	167	38	24
5	Ig heavy chain precursor	gi|108750	51 391	6.1	251	7	23
5	Ig lambda light chain	gi|15088675	25 032	5.84	132	4	20
5	Alpha-S1-casein isoform X2	gi|982928492	23 558	5.12	111	4	21
6	Ig lambda-like polypeptide 1	gi|741957421	25 010	8.19	457	10	44
6	Ig light chain, lambda gene cluster	gi|92096965	24 863	7.53	449	9	37
7	Alpha-S1-casein	gi|225632	24 477	4.85	665	8	40
7	Beta-lactoglobulin	gi|2194088	18 583	4.83	115	6	32
8	Beta-casein isoform X1	gi|741930202	29 150	5.89	305	35	44
8	Component PP3	gi|741536	15 295	5.98	144	4	26
9	Beta-lactoglobulin	gi|229460	18 641	4.76	163	6	48
9	Alpha-S1-casein isoform X13	gi|528953246	20 227	5.32	140	4	28
10	Beta-lactoglobulin	gi|6980895	18 641	4.76	2325	18	82
11	Alpha-lactalbumin	gi|68	*14* *603*	4.8	392	4	39
11	Beta-lactoglobulin	gi|2194088	18 583	4.83	308	8	48

### 3.2 Acute phase proteins

**Fig. 2:**
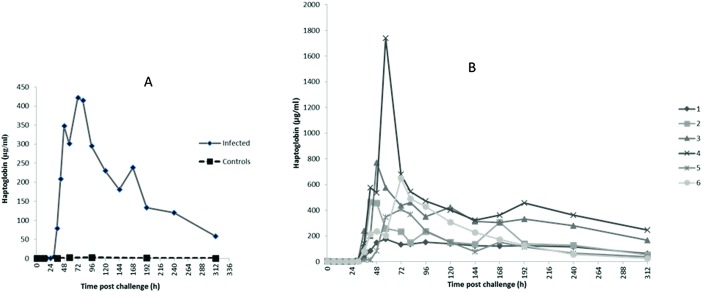
Haptoglobin concentration in bovine mammary quarters challenged with *Streptococcus uberis* (infected, *n* = 6) or mock challenged with phosphate buffered saline (controls, *n* = 6). Results show median (A) and individual (B) concentrations.

**Fig. 3:**
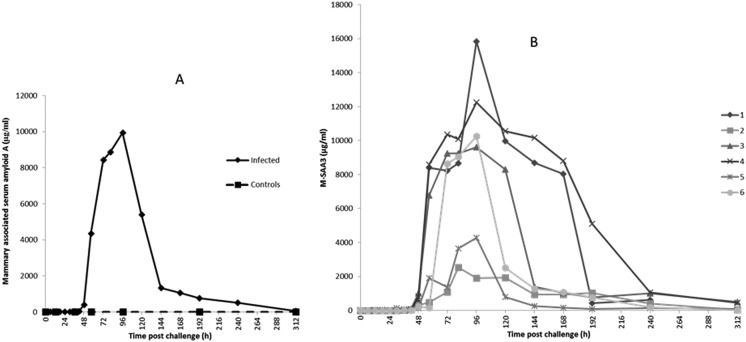
Mammary associated SAA3 concentration in bovine mammary quarters challenged with *Streptococcus uberis* (infected, *n* = 6) or mock challenged with phosphate buffered saline (controls, *n* = 6). Results show median (A) and individual (B) concentrations.

**Fig. 4:**
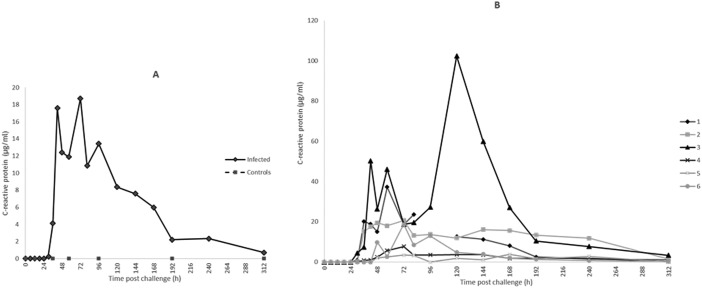
C-Reactive protein concentration in bovine mammary quarters challenged with *Streptococcus uberis* (infected, *n* = 6) or mock challenged with phosphate buffered saline (controls, *n* = 6). Results show median (A) and individual (B) concentrations.

The profiles of Hp, M-SAA3 and CRP over time during the *S. uberis* mastitis challenge are shown in Fig. 2, 3 and 4 respectively with the median value and the individual values shown for the six infected quarters from cows 1, 2, 3, 4, 5, 6 (cow numbers consistent with Tassi *et al.* ^4^).

The earliest rise in Hp concentration was seen at 36 h PC with concentrations over 100-fold the median for pre-challenge (0 h PC) observed in 4 challenged quarters, and with all samples reaching this level by 48 h PC. The maximum median concentration of 421 μg ml^–1^ (Fig. 2A) was observed at 72 h PC. At the final time of sampling (312 h), two quarters still had elevated Hp concentrations relative to basal values (cow 3 and 4 in Fig. 2B). In control samples (*n* = 42), the range of Hp concentration was <0.4–6.38 μg ml^–1^, and in pre-challenge samples (0 h, *n* = 6) it was <0.4–1.26 μg ml^–1^.

The first rise in M-SAA3 levels was also observed at 36 h PC with 5 of 6 milk samples showing at least a 20-fold increase over the median of the 0 h PC samples and with all samples showing more than a 100-fold the 0 h PC median by 48 h PC. The maximum median concentration of M-SAA3 was at 96 h was 9900 μg ml^–1^ (Fig. 3A). At 312 h, two quarters had high M-SAA3 concentration; these two quarters were the same ones which had higher Hp concentration at 312 h (cow 3 and 4, Fig. 3B). A range of <0.6–18.68 μg ml^–1^ was found in control samples and <0.6–19.22 μg ml^–1^ in pre-challenge samples (0 h).

For CRP, the first rise in concentration in milk was at 30 h PC with 3 of 6 samples at least 300× the 0 h PC median concentration and with all samples having over 1000× the value at 48 h PC. Peak median concentrations of CRP were achieved at 72 h at 16 687 ng ml^–1^ (Fig. 4A). At 120 h PC there was a peak of CRP in cow 3 at 102 000 ng ml^–1^ while at 240 h CRP concentrations in cows 2 and 3 were noticeably higher than in the other cows (Fig. 4B). The range of CRP in control samples was <1.8–41.44 ng ml^–1^ and was <1.8 ng ml^–1^ in pre-challenge samples.

### 3.3 IMI77 classifier based on CE-MS datasets

In order to detect IMI in cows, CE-MS datasets from 6 cows were analysed. According to specific guidelines on biomarker studies,^36^ samples were split into the discovery cohort formed by 12 samples, 6 samples from 0 h PC (non infected, NI) and 6 infected cows from 81 h PC (infected, I). The validation cohort consisted of 23 milk samples collected at 36, 42, 57 and 312 h PC (for all time points *n* = 6, except for 36 h PC where *n* = 5 as there was insufficient volume for one sample).

**Fig. 5:**
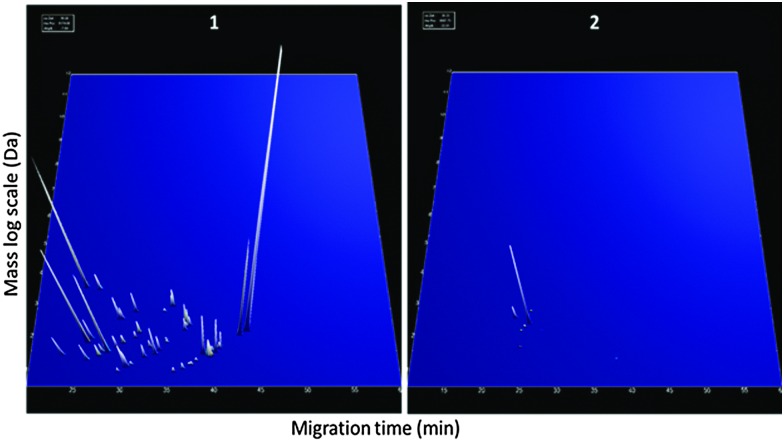
Peptides detected in milk fluid and differences between non-infected cows (0 h PC) and infected (36, 42, 57 and 81 h PC). Representation of the up-regulated (left panel) and down-regulated (right panel) peptides analysed by CE-MS. Each peptide was identified by a unique identifier based on the migration time (min) and specific mass (kDa), with a peak height representing the relative abundance.

**Fig. 6:**
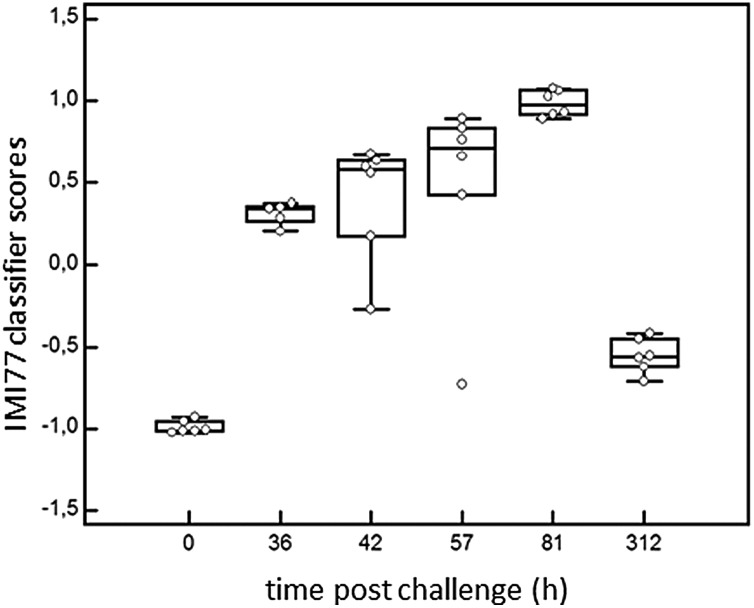
Performance of the classifier in the discovery cohort (0 and 81 hours) and progression of infection (36 h, 42 h, 57 h, 312 h PC). Box whisker plot according to the IMI77 score showing median, 10th, 25th, 75th and 90th percentiles.

Comparison of the peptide profiles from the two sets of samples in the discovery cohort led to the identification of 460 peptides with adjusted BH *p*-value significant (*P* < 0.05) that were present in at least 66% of the control or diseased groups. Those displaying an AUC = 1 were further considered for the study (205 peptides). LC-MS/MS analysis, and data matching with those from Mansor *et al.* ^11^ allowed 77 sequences to be obtained from these 205 peptides (Table 2). Peptide maps (CE-MS peaks) of potential biomarkers of *S. uberis* mastitis which were up-regulated or down regulated during infection at 36, 42, 57 and 81 h PC relative to 0 h (pre-challenge) are shown in Fig. 5. Out of the 77 peptides, 50 showed qualitative differences between the 0 and 81 h PC (being totally absent at one time as against the other), and 27 displayed quantitative changes with the course of infection. Fifty-five polypeptides were increased in abundance. Among them, the most abundant fragments corresponded to proteins such as alpha-S1-casein and alpha-S2-casein (36 peptides), beta-casein (22 peptides), serum amyloid and Glycosylation-dependent cell adhesion molecule 1 (GDCAM) (5 peptides each). The 77 sequence peptides were then used in a support vector machine (SVM) classifier called IMI77. After applying cross-validation of the discovery data, no peptide was left out from the final classifier. Scoring the animals from the discovery cohort with the resulting IMI77 classifier clearly separated non infected cows from the infected ones. In the next step, the classifier was applied to the 23 samples that were not used in the discovery cohort to see its performance in the progression of IMI. The distribution of IMI77 scores for the discovery and validation cohort showed a pattern where the score increased with the time of infection up to 81 h PC but with samples from 312 h PC the score was more comparable to control than infected animals (Fig. 6).

**Table 2:** Peptides used in IMI77 classifier. Frequency and intensity indicate the number of samples in which each peptide was detected/number of quarter milk samples per group (*n* = 6) and the average ion counts, respectively for samples collected pre-challenge (0 h PC, non infected, NI) and at 81 h PC (infected, I)

Peptide_ID	Protein symbol	Protein name	Sequence	Frequency NI	Intensity NI	Frequency I	Intensity I	Fold change I/NI	Direction I/NI
5003	GLCM1	Glycosylation-dependent cell adhesion molecule 1	SHAFEVVKT	2/6	1.7	6/6	599.2	344.3	Up
5320	CASA1	Alpha-S1-casein	QQKEPMIGV	1/6	0.4	6/6	692.4	1731.0	Up
7162	CASA2	Alpha-S2-casein	QKFALPQYL	1/6	3/6	6/6	968.6	1793.8	Up
8859	CASB	Beta-casein	SEESITRINK	0	0	6/6	2194.7	2194.7	Up
8906	CASA1	Alpha-S1-casein	NELSKDIGSES	6/6	154.2	0	0	0	Down
9741	CASB	Beta-casein	YPQRDMPIQA	1/6	6/6	6/6	297.0	309.4	Up
9931	LACB	Beta-lactoglobulin	EELKPTPEGDL	1/6	1/6	6/6	445.2	2473.3	Up
10197	CASA1	Alpha-S1-casein	HAQQKEPMIGV	0	0	6/6	528.1	528.1	Up
10508	CASA2	Alpha-S2-casein	TKVIPYVRYL	6/6	1852.2	0	0	0	Down
12245	CASB	Beta-casein	LSSSEESITRIN	0	0	6/6	433.0	433.0	Up
13263	CASA1	Alpha-S1-casein	HPIKHQGLPQEV	2/6	31.1	6/6	2295.1	73.7	Up
13326	CASA1	Alpha-S1-casein	IPNPIGSENSEKT	6/6	671.8	2/6	17.5	0	Down
14354	CASA1	Alpha-S1-casein	VAPFPEVFGKEKV	1/6	22.3	6/6	1952.1	87.5	Up
14551	CASA1	Alpha-S1-casein	YKVPQLEIVPNSA	1/6	4.9	6/6	412.3	85.0	Up
15151	CASB	Beta-casein	AVPYPQRDMPIQA	0	0	6/6	1440.3	1440.3	Up
15287	CASA1	Alpha-S1-casein	FVAPFPEVFGKEK	6/6	343.6	0	0	0	Down
15326	CASB	Beta-casein	EMPFPKYPVEPF	0	0	6/6	2261.4	2261.4	Up
15395	CASA1	Alpha-S1-casein	DIPNPIGSENSEKT	0	0	6/6	416.3	416.3	Up
15403	CASA1	Alpha-S1-casein	HIQKEDVPSERY	1/6	44.4	6/6	1591.8	35.8	Up
15580	CASA1	Alpha-S1-casein	KHPIKHQGLPQEV	0	0	6/6	2616.1	2616.1	Up
15923	LACB	Beta-lactoglobulin	SLLDAQSAPLRVYV	1/6	0.6	6/6	5381.2	9277.9	Up
16011	CASA1	Alpha-S1-casein	EGIHAQQKEPMIGV	0	0	6/6	3192.3	3192.3	Up
16211	CASA1	Alpha-S1-casein	EGIHAQQKEPmIGV	0	0	6/6	667.2	667.2	Up
16353	SAA	Serum amyloid A protein	GNYDAAQRGPGGAWAA	1/6	61.7	6/6	2005.7	32.5	Up
16692	CASA1	Alpha-S1-casein	SDIPNPIGSENSEKT	0	0	6/6	4668.6	4668.6	Up
16863	CO3	Complement C3	SEETKENERFTVK	3/6	9.0	6/6	1244.5	138.3	Up
17132	CASA1	Alpha-S1-casein	HIQKEDVPSERYL	1/6	11.9	6/6	9426.7	795.5	Up
17453	CASB	Beta-casein	AVPYPQRDMPIQAF	0	0	6/6	754.6	754.6	Up
17789	SAA	Serum amyloid A protein	GADKYFHARGNYDAA	0	0	6/6	1326.7	1326.7	Up
17818	OSTK	Osteopontin-K	IRISHELDSASSEVN	0	0	6/6	2132.0	2132.0	Up
18670	CASA1	Alpha-S1-casein	NELSKDIGSESTEDQA	0	0	6/6	924.8	924.8	Up
18956	CASB	Beta-casein	QKAVPYPQRDMPIQA	0	0	6/6	1016.0	1016.0	Up
19009	CASB	Beta-casein	HKEMPFPKYPVEPF	0	0	6/6	1767.1	1767.1	Up
19028	CASB	Beta-casein	HKEMPFPKYPVEPF	6/6	2655.3	4/6	153.2	0.1	Down
19318	CASB	Beta-casein	FPKYPVEPFTESQSL	1/6	8.4	6/6	2113.3	251.3	Up
19331	CASA2	Alpha-S2-casein	LYQGPIVLNPWDQVK	6/6	376.6	0	0	0	Down
20271	SAA	Serum amyloid A protein	RGNYDAAQRGPGGAWAAK	0	0	6/6	632.3	632.3	Up
20714	CASA1	Alpha-S1-casein	SMKEGIHAQQKEPMIGV	0	0	6/6	1119.7	1119.7	Up
20789	CASB	Beta-casein	QKAVPYPQRDMPIQAF	0	0	6/6	403.3	403.3	Up
20919	SAA	Serum amyloid A protein	SGKDPNHFRPAGLPDKY	0	0	6/6	10457.3	10457.3	Up
21739	CASK	Kappa-casein	SRYPSYGLNYYQQKPV	0	0	6/6	270.8	270.8	Up
22168	CASA1	Alpha-S1-casein	EQKHIQKEDVPSERYL	0	0	6/6	2672.9	2672.9	Up
22421	CASB	Beta-casein	QKAVPYpQRDMPIQAFL	0	0	6/6	935.6	935.6	Up
23769	CASA1	Alpha-S1-casein	GIHAQQKEPMIGVNQELAY	2/6	34.7	6/6	10893.4	313.7	Up
24045	GLCM1	Glycosylation-dependent cell adhesion molecule 1	SSRQPQSQNPKLPLSILKE	6/6	817.9	0	0	0	Down
24098	FGFP1	Fibroblast growth factor-binding protein 1	RGSKASADESLALGKPGKEPR	6/6	661.2	0	0	0	Down
24482	CASA2	Alpha-S2-casein	TMEHVSSSEESIISQETYK	0	0	6/6	1541.8	1541.8	Up
24847	CASA1	Alpha-S1-casein	SDIPNPIGSENSEKTTMPLW	6/6	703.8	6/6	20422.2	29.0	Up
25003	CASA1	Alpha-S1-casein	SDIPNPIGSENSEKTTmPLW	1/6	3.5	6/6	5216.3	1481.9	Up
25030	CASA1	Alpha-S1-casein	RPKHPIKHQGLPQEVLNEN	6/6	2951/6	2/6	111.4	0	Down
25054	CASA1	Alpha-S1-casein	HPIKHQGLPQEVLNENLLR	6/6	918.0	1/6	9.8	0	Down
25195	CASB	Beta-casein	VLPVPQKAVPYPQRDMPIQA	0	0	6/6	1612.7	1612.7	Up
25582	GLCM1	Glycosylation-dependent cell adhesion molecule 1	SSRQPQSQNPKLPLSILKEK	6/6	6164.4	0	0	0	Down
25911	CASA2	Alpha-S2-casein	KNTMEHVSSSEESIISQETY	6/6	1199.6	0	0	0	Down
26545	CASA1	Alpha-S1-casein	RPKHPIKHQGLPQEVLNENL	6/6	3084.5	4/6	293.0	0.1	Down
26799	CASB	Beta-casein	WMHQPHQPLPPTVmFPPQSV	0	0	6/6	576.5	576.5	Up
27098	CASB	Beta-casein	VLPVPQKAVPYPQRDMPIQAF	0	0	6/6	1251.4	1251.4	Up
27560	CASA1	Alpha-S1-casein	HIQKEDVPSERYLGYLEQLL	6/6	2623.0	0	0	0	Down
27692	CASB	Beta-casein	SWMHQPHQPLPPTVMFPPQSV	0	0	6/6	2676.3	2676.3	Up
27904	GLCM1	Glycosylation-dependent cell adhesion molecule 1	ILNKPEDETHLEAQPTDASAQF	6/6	695.2	0	0	0	Down
27994	CASA1	Alpha-S1-casein	RPKHPIKHQGLPQEVLNENLL	6/6	52176.1	5/6	8867.9	0.2	Down
28202	CASB	Beta-casein	FQSEEQQQTEDELQDKIHPF	0	0	6/6	1862.6	1862.6	Up
28876	GLCM1	Glycosylation-dependent cell adhesion molecule 1	SSRQPQSQNPKLPLSILKEKHL	6/6	22515.4	0	0.0	0.0	Down
29718	CASB	Beta-casein	QSKVLPVPQKAVPYPQRDMPIQA	0	0	6/6	1521.7	1521.7	Up
29972	SAA	Serum amyloid A protein	GADKYFHARGNYDAAQRGPGGAWAA	0	0	6/6	3021.4	3021.4	Up
30120	CASA1	Alpha-S1-casein	RPKHPIKHQGLPQEVLNENLLR	6/6	15286.8	5/6	1592.0	0.1	Down
31513	CASA1	Alpha-S1-casein	LKKYKVPQLEIVPNSAEERLHSM	6/6	389.1	0	0.0	0.0	Down
32317	LACB	Beta-lactoglobulin	RTPEVDDEALEKFDKALKALPMHI	6/6	1491.6	0	0.0	0.0	Down
32654	CASA1	Alpha-S1-casein	EERLHSMKEGIHAQQKEPMIGVNQ	6/6	3355.6	0	0.0	0.0	Down
33130	CASB	Beta-casein	MAPKHKEMPFPKYPVEPFTESQSL	0	0	6/6	2502.4	2502.4	Up
33228	CASB	Beta-casein	SQSKVLPVPQKAVPYPQRDMPIQAF	0	0	6/6	376.7	376.7	Up
33323	CASA1	Alpha-S1-casein	HIQKEDVPSERYLGYLEQLLRLK	6/6	6037.7	1/6	115.9	0.0	Down
35775	CASB	Beta-casein	LSLSQSKVLPVPQKAVPYPQRDMPIQA	1/6	5.9	6/6	1353.1	230.1	Up
36371	CASB	Beta-casein	SLSQSKVLPVPQKAVPYPQRDMPIQAF	0	0	6/6	2161.4	2161.4	Up
44033	CASK	Kappa-casein	TMARHPHPHLSFMAIPPKKNQDKTEIPTINT	0	0	6/6	1822.2	1822.2	Up
44930	TRBP2	RISC-loading complex subunit TARBP2	GWRLPEYTVTQESGPAHRKEFTMTCRVERF	0	0	6/6	12223.3	12223.3	Up
1217454	CASA1	Alpha-S1-casein	FPEVFGKEKV	1/6	4.0	6/6	3105.0	770.5	Up

### 3.4 Liquid chromatography and mass spectrometry

Liquid chromatography-tandem mass spectrometry allowed for sequencing of the 77 peptides in the biomarker panel which were matched with 3 multi-consensus reports and a report of Mansor *et al.* ^11^ Along with some of their characteristics, they are listed in Table 2. Mass to charge ratio (*m*/*z*) range of the sequenced peptides was from 498.93 to 1008.88 Da and mass range from 1016.5 to 3610.74 Da. Most of the sequenced peptides arose from cleavages of alpha-S1-casein and other caseins. A few were from SAA and GDCAM proteins. Some of the peptides derived from SAA protein were up regulated by several thousand folds during peak of infection, for example; GADKYFHARGNYDAA, GADKYFHARGNYDAAQRGPGGAWAA and SGKDPNHFRPAGLPDKY.

The greatest fold change (12 223×) occurred with the polypeptide GWRLPEYTVTQESGPAHRKEFTMTCRVERF which had sequences matching into the RISC-loading complex subunit protein. This peptide was the most up regulated peptide identified followed by SGKDPNHFRPAGLPDKY derived from SAA protein (10 457×). There were 22 peptides which were down regulated among the total 77 sequenced and these were derived mainly from alpha-caseins and GDCAM proteins.

## 4. Discussion

**Fig. 7:**
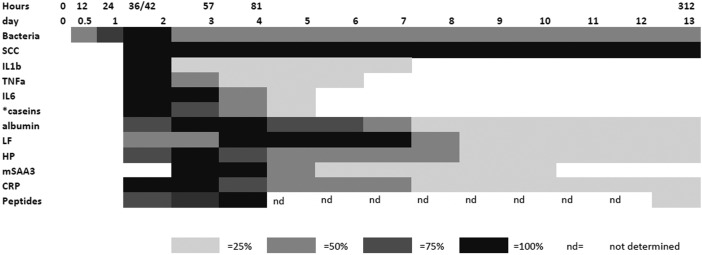
The relative responses of analytes following experimental infections with *S. uberis* combining results from this investigation and those described by Tassi *et al.* ^4^ The shading represents increasing responses in relation to the peak response and represents 25%, 50%, 75% and 100% of peak response on days PC. Responses were increased from the day 0 levels except where indicated by * which were decreases with respect to the day 0 level.

**Fig. 8:**
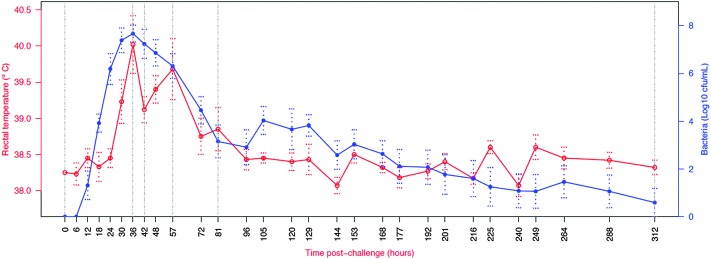
Course of infection in challenged cows (*n* = 6) as indicated by average body temperature and average bacterial count in milk. Number of culture positive quarters ranged from six at 18 to 72, 105 and 129 h post challenge to one at 312 h post challenge.^4^ Vertical lines indicate time points for which peptidomic analysis was conducted. Normally IMI definition is based on the presence of bacteria in milk samples, whereby three consecutive negative samples are needed to declare an animal free of IMI.

In order to integrate the results on the high abundance proteins, the APP and peptides in milk in relation to changes already described by Tassi *et al.*,^4^ Fig. 7 shows the change in selected analyte levels from the current and the previous studies based on the percentage of the maximal increase for each. To further enable interpretation and integration of data Fig. 8 shows the mean bacterial count and rectal temperatures of the infected cows as previously described.^4^ Bacterial count in milk was the first parameter to increase being observed at 12 h PC, reaching a peak at 36 h PC and falling to around 50% of peak bacteria from 72 h PC to the end of study. It should be noted that IMI would normally be defined based on the presence of bacteria in milk samples, whereby three consecutive negative samples are needed to declare an animal free of IMI. The SCC first increased at 30 h PC, reached a peak at 48 h PC and plateaued at this level virtually to the end of the study. Among the cytokines, IL1β, TNFα and IL6 reached peaks between 36–72 h PC and declined to low levels by 120 h PC.

### 4.1 High abundance proteins of milk

The IMI with *S. uberis* caused significant change in the high abundance milk proteins and increases in milk APP. While there was between animal variations in the response of high abundance proteins to IMI, there were consistent changes seen along the time course of the infection in the sets of milk samples from each infected udder quarter. The decrease in caseins, β-lactoglobulin and α-lactalbumin and increase in albumin, LF and IgG following infection of the mammary are well known^18,19,29^ but here the timing of the responses has been identified. With cow 6 (Fig. 1) as an example the fall in caseins of 28–38 kDa was seen first at 36 h PC, occurring after bacterial count and SCC increases which were at 12 h and 30 h PC respectively but at the same time as increases in cytokines such as TNFα and IL1β.^4^ There was a subsequent increase in the protein at 28–38 kDa from 72 h PC but in mastitic milk (Fig. 1B) Ig light chain has a similar mobility and with one dimensional electrophoresis it is not possible to differentiate between these proteins. Two dimensional electrophoresis or immunoassay would be needed to achieve this purpose. Increases in albumin and IgG occurred later, at 81 h PC, while the peak of LF was further delayed to 120 h PC. Thus changes in the concentrations of high abundance proteins of milk following IMI are not uniform across proteins. It may be that, by monitoring relative concentrations of these proteins, alone or as part of a diagnostic panel, the stage of infection could be identified. Although IMI had been resolved in 5 of 6 animals by 312 h PC,^3^ the composition of high abundance proteins had not reverted to pre-challenge levels. By contrast, SCC levels were still high in all cows at 312 PC,^4^ implying that host rather than bacterial proteases are responsible for protein degradation.

### 4.2 Acute phase proteins

For the APP, the time course of increase in Hp and M-SAA3 have been described in response to *S. aureus* mastitis^10^ but the changes in milk CRP during any experimental model of mastitis have not previously been demonstrated. In respect to the cytokine response the maximum of Hp, M-SAA3 and CRP concentration were after the peak cytokine responses (Fig. 7).

There was variation between the individual cows in APP response, as there had been in clinical and bacteriological response.^4^ Milk Hp was first increased from basal values at 36 h PC in 4 cows with the median Hp across all cows peaking at 72 h PC. Notably, over several hundred fold increase in milk Hp concentration was observed at the peak, highlighting the strong response of milk Hp to the IMI. Elevation of milk CRP was the earliest to occur with the initial increase being observed at 30 h pi in 3 out of the six cows while M-SAA3 was the last to be raised with only a 20× increase seen at 36 h PC. There were differences between animals as well as between the APP, but the APP responses were consistent in a number of aspects. At least 24 h passed between infection and any elevation of the milk APP concentration. The APP showed over a thousand fold increase in their concentrations with maximum median concentrations at 72–96 h PC, thereafter falling though in some cows the basal level of the APP had not been reached by 312 h PC. The fall in APP after 72 h PC occurred even though the SCC remained elevated for the duration of the 312 h of the study and in the resolution phase more closely resembled the profile of bacterial counts in the milk than the SCC. Hence, APP may be a better biomarker of IMI than SCC.

There are differences to previous reports on APP in mastitis. Pedersen and others (2003)^23^ and Jacobsen *et al.* (2005)^37^ demonstrated an earlier rise in M-SAA3 than Hp during the course of an *S. uberis* intramammary challenge. The difference in comparison to our results could be due to strain differences in the *S*. *uberis* used for challenge leading to different cytokine activation pathways^16^ and could also be influenced by a difference in host genotype or phenotype. While assays for bovine CRP have only recently become available it could be that using such an analyte with a lower detection limit and a large dynamic range will accentuate the value of this APP in detecting mastitis. Previously, although CRP had been identified as a milk APP^24^ it has not generally been regarded as a bovine APP for use as a biomarker of mastitis, but availability of the immunoassay used here for bovine CRP will allow its diagnostic value to be assessed at a larger scale. Currently, of the three APP, Hp is the easiest to measure with availability of specific antibody for the development of varied immunoassay formats and a large response even if its peak response is later than that of CRP. The stage of IMI and the species of pathogen are known to cause differing mammary responses.^38,39^ While attempts to differentiate pathogen and stage of IMI by APP analysis have yielded disappointing results^40^ an aim of the current series of studies is to determine whether differentiation is possible with inclusion not only of Hp, MSAA3 and CRP but also change in the high abundance proteins, peptides and metabolites possibly yielding a diagnostic algorithm similar to those being developed for protein profiles being developed in clinical proteomics^41^ and could yield diagnostic value for mastitis detection and monitoring.

### 4.3 Peptidomics

A limitation of previous investigations of the responses of milk proteins to mastitis has been that, due to the lack of suitable methods, the low *M*_w_ proteins and peptides in milk are frequently ignored. Recently the use of methods specific for peptides of <25 kDa have suggested that there are major changes in these molecules in mastitis. CE-MS analysis of bovine milk during natural mastitis^11^ detected peptide differences between milk samples from control and naturally infected udders (31 polypeptides) and between milk from mastitic udders caused by two separate pathogens (14 polypeptides). This method of peptide analysis has been described as a powerful hyphenated technique for the study of peptidomic profiles^42^ and has been exploited for the generation of biomarker panel of peptides for conditions such as renal^43^ and cardiovascular^44^ disorders in humans.

A majority of the successfully sequenced changing peptides from this challenge study arose from cleavages of alpha-S1-casein (*n* = 31) and beta-casein (22 milk proteins), in agreement with the reports of Dallas *et al.* (2014),^45^ Mansor *et al.* (2013)^11^ and Larsen *et al.* (2010)^46^ and despite differences in causative agents between studies. This further explains the general decrease in milk caseins associated with clinical mastitis^20^ and shown here in Fig. 1. It has been postulated that *S. uberis* is dependent on casein cleavage to obtain nutrients during IMI,^47^ but shifts in protein and peptide distributions persist beyond resolution of infection so casein cleavage is not dependent on the pathogen.

A few of the peptides showing change were not from casein degradation but from GDCAM, (mainly down regulated), and SAA (up regulated) cleavages. These two proteins have been identified as immune related proteins.^29,48,49^ Presence of GDCAM could relate to the role proposed for host glycosaminoglycans in the pathogenesis of *S. uberis* mastitis.^50,51^ Proteases play a central role in the type and amounts of peptides detected in milk during mastitis and endogenous peptides such as plasmin, cathepsins, elastase, and amino- and carboxypeptidases have been suggested as being crucial during the IMI as they are increased in milk due to release from the influx of neutrophils (PMNs) and other phagocytic cells, measured as the SCC, that occurs during mastitis.^46,52^ These proteases were also reported to have specificities towards alpha-S1 and beta caseins. Pathogen related proteases have also been suggested to contribute to the proteolysis observed in milk during mastitis.^46^

Similar to the study of Wedholm *et al.* (2008),^53^ peptides from alpha-S1, alpha-S2 and beta-caseins were identified but in addition two kappa-caseins fragments were found and sequenced during infection but were absent in pre-challenge samples. This corresponds to the effect of LPS infusion in an experimental mastitis model generating proteolytic changes of milk over time.^52^

Three polypeptides sequenced in this study were similarly identified in both the multi-consensus and Mansor *et al.* (2013)^11^ reports. Two of these peptides were fragments from GlyCAM-1 protein and one was from cleavage of fibroblast growth factor-binding protein (FGFBP). All of these three polypeptides were found in pre-challenge samples and absent during infection, while in the study of Mansor *et al.* (2013),^11^ these polypeptides only differentiated between healthy and mastitic samples and not between the two different mastitis pathogen species studied (*i.e. E. coli* and *S. aureus*). The matching of these peptides from the present study, the study of Mansor *et al.* (2013)^11^ and with reports from previous CE-MS milk analysis substantiates their probability as peptide markers of mastitis irrespective of the causal agent of mastitis.

As a time-point-based peptidomic study of mastitis progression, this study offers additional advantage over other previous investigations in detecting and identifying peptides and in showing significant difference from pre-challenge controls, as early as 36 h PC. The probability exists that the peptidomic profile at earlier time points (before 36 h) may significantly differentiate pre-challenge samples from commencement of infection but were not analysed here due to resource limitations. As an objective for future studies, it would be useful to determine the earliest time point during which peptide changes are able to significantly differentiate healthy from infected samples to provide an early warning of impending mastitis.

The increase in IMI77 classification score up to 81 h PC shows that peptide proteolysis increases while the bacterial count declined after 30 h PC. The proteolytic activity may thus be more likely to be emanating from endogenous proteases rather than those of bacterial origin. It was of interest that at 81 h PC there were no peptides derived from albumin, lactoferrin or IgG despite these being the most abundant proteins in the milk at this time point. These proteins may be more resistant to degradation by the proteases present in the milk than the caseins. This could be a part of an anti-bacterial function of the alteration of the milk proteome in mastitis by depriving bacteria of protein as a nutrient but still providing protein in the milk that would be digested by the neonate’s gastro-intestinal tract.

In respect of a peptide panel that could differentiate mastitis caused by *S. uberis* from other pathogens, 72 of the polypeptides which were sequenced in this study, did not match any of the polypeptides detected in Mansor *et al*.’s study^11^ of *S. aureus* and *E. coli* mastitis or any of the multi-consensus reports. Therefore, these 72 peptides could represent a panel of peptides specific to *S. uberis* mastitis. Validation of this claim would be required using other *S. uberis* mastitis models such as natural infection and infections by different strains of *S. uberis.*

The time points that were selected for peptidomic analysis were based on the clinical and bacteriological course of infection, whereby the peak of infection seemed to have ended by 81 h post challenge (Fig. 8). Surprisingly, the biggest peptidomic difference between pre-challenge and post-challenge samples was detected in the validation set, using samples from 81 h post challenges. Indeed, changes in high abundance proteins, APP and peptidomic profiles all persisted beyond the clinical and bacteriological peak of IMI, indicating that bacteriological, clinical and peptidomic events are partly out of synch. This is consistent with the idea that changes in proteins and peptides are largely driven by the host immune response and SCC influx rather than directly by bacteria. At the last observed time point, 312 h PC, the IMI77 classifier scores were still significantly different from the pre-challenge time point, but much closer to pre-challenge values than for any other time point considered in this study. At 312 h PC, 5 of 6 cows had resolved the IMI and all cows and quarters appeared clinically normal.^3^ Thus, the change in IMI77 score reflects the natural resolution of IMI. It would be interesting to explore the relationship between bacteriological status and peptide profile at individual cow level for multiple time points during the IMI resolution phase but samples to do so were not available from the current study.

Early detection and differential diagnosis of the mastitis causing pathogen would be valuable for the dairy industry, for earlier and more effective treatment and also to reduce the use of ineffectual antimicrobials which would lead to a reduction in resistance to these therapeutics. On large dairy farms operating under high economic pressure and on farms with automated milking systems, clinical symptoms would not be noticed because regular observation of individual animals does not take place. Under those circumstances, alternative diagnostic indicators are potentially of great value. It is clear that both APP and peptide analysis could play a role in this scenario and when combined with quantitative proteomics^12^ and metabolomics,^13^ that integration of protein assay and omic technologies has major potential for delivering a unified and substantial means to provide a molecular insight into a complex biological system and to stimulate biomarker development across omic boundaries.

## 5. Conclusion

The high abundance protein and APP profiles of milk during an experimental *S. uberis* mastitis challenge were investigated, with a shift in abundance from caseins, β-lactoglobulin and α-lactalbumin to albumin, lactoferrin and IgG being observed following infection. The APP profiles of Hp, M-SAA3 and CRP were closer to the bacterial count than the SCC in milk from infected quarters and may have value in diagnosing and monitoring the stage of IMI. Analysis of the peptide profile in milk across selected time points of the experimental challenge, showed a panel of peptides, which as early as 36 h PC, could significantly differentiate infected from non-infected milk, thus suggesting potential as biomarkers of bovine mastitis. Moreover, the identification of peptidomic markers that were not detected in clinical mastitis due to other pathogens suggests that pathogen specific diagnosis is possible.
