# Supplementary material for: Mastitomics, the integrated omics of bovine milk in an experimental model of Streptococcus uberis mastitis: 1. High abundance proteins, acute phase proteins and peptidomics
Source: Mol Biosyst. 2016 Jul 14;12(9):2735–47. doi: 10.1039/c6mb00239k (PMC5048397; doi:10.1039/c6mb00239k)
Supplement: Supplementary file 1 [file MB-012-C6MB00239K-s001.pdf]

Electronic Supplementary Material (ESI) for Molecular BioSystems.
This journal is © The Royal Society of Chemistry 2016

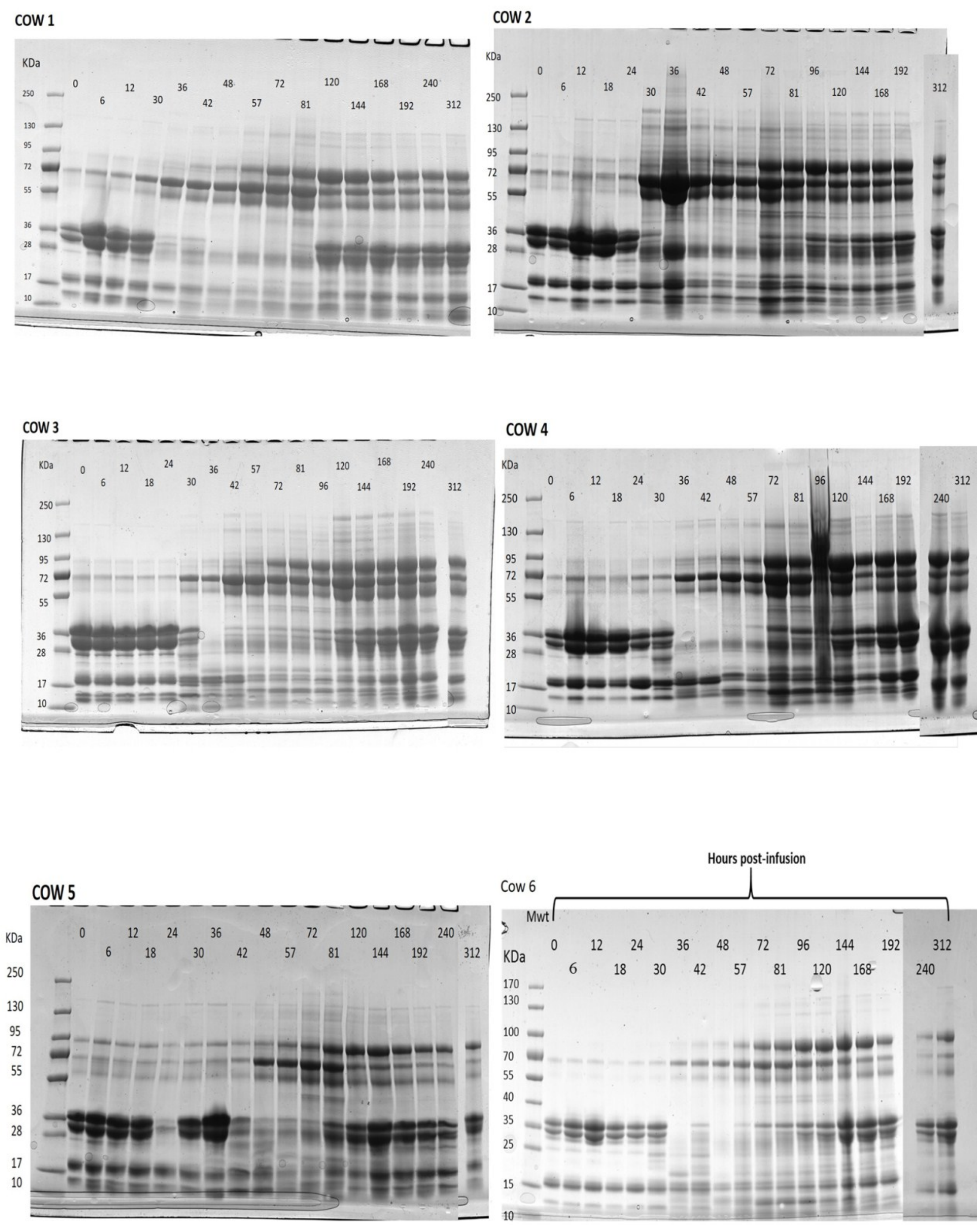

Figure S1 One dimensional SDS-PAGE gels showing high abundance proteins from mammary quarters of cows 1-6 challenged with *Streptococcus uberis.* Each gel has Mw markers in kDa in the left hand track. Milk samples are from 0, 6, 12, 18, 24, 30, 36, 42, 48, 57, 72, 81, 96, 120, 144, 68, 192, 240 and 312 h PC in gels where they were available but occasional time point samples were omitted where sample volume was insufficient for analysis.
